# To Attract Others, Immune Cells Release a Packet Which Releases a Signal

**DOI:** 10.1371/journal.pbio.1002337

**Published:** 2016-01-07

**Authors:** Richard Robinson

**Affiliations:** Freelance Science Writer, Sherborn, Massachusetts, United States of America

When you cut your finger, molecules released by damaged cells (and later, by growing bacteria) are sensed by roving immune cells called neutrophils, which respond to the signal by moving directly and rapidly toward the site of damage. They also release their own set of chemicals, especially leukotriene B_4_ (LTB_4_), essentially signaling to other neutrophils, “Hey, follow me!” The release of LTB_4_ both amplifies the primary damage signal and casts it more widely, bringing in far more reinforcements than the damaged cells themselves could muster on their own.

The neutrophil knows which direction it should go by following the signal up its concentration gradient. However, it has been unclear how a diffusible secondary attractant such as LTB_4_ can be disseminated far from where it is released, and thereby amplify the diffusible primary attractants and recruit more neutrophils to the site of injury and inflammation. In a new study in *PLOS Biology*, Ritankar Majumdar, Aidin Tavakoli Tameh, and Carole Parent show that neutrophils release not the LTB_4_ itself but vesicles that contain it along with the enzymatic machinery to make it, allowing LTB_4_ to be dispersed over larger areas and for longer time periods than through direct release (Fig 1).

**Fig 1:**
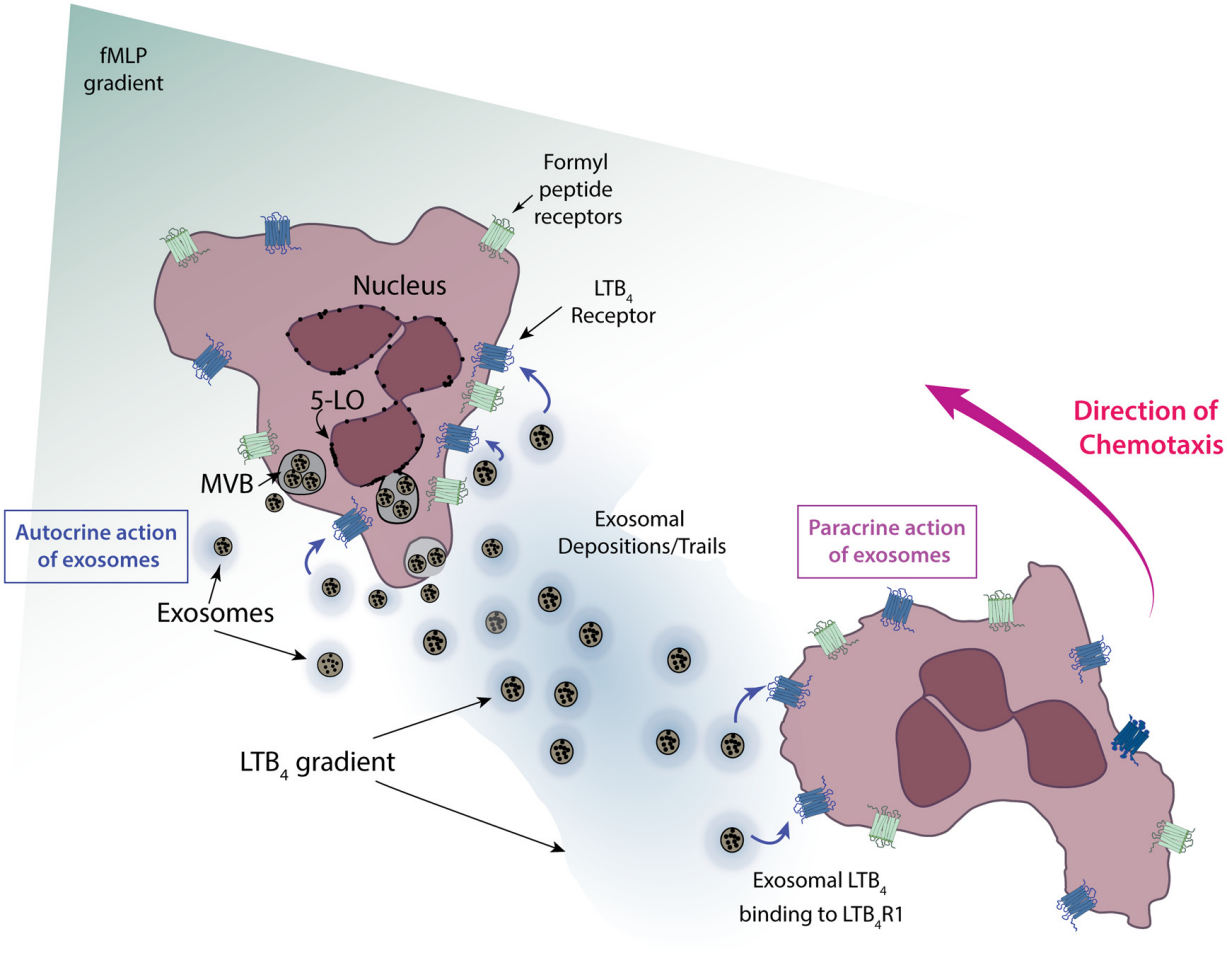
Cartoon depicting the events involved in the relay of primary chemotactic signals to the secondary chemoattractant LTB_4_ during neutrophil chemotaxis. Image Credit: Ritankar Majumdar.

Neutrophils release large amounts of LTB_4_ only upon activation by a primary chemoattractant, such as N-formylmethionyl-leucyl-phenylalanine (fMLP), released by both damaged cells and bacteria. The authors began by activating neutrophils and then separating their subcellular components by density. They found that LTB_4_ was not increased in the density fractions associated with the most common secretory pathways, but its distribution pattern did match that for a protein known to be packaged in multivesicular bodies (MVBs).

As their name implies, MVBs are membrane-bound structures that contain numerous smaller vesicles (called intraluminal vesicles). When an MVB fuses with the plasma membrane, its intraluminar vesicles are released into the extracellular space (at which point they are called exosomes). Using electron microscopy, the authors found that one of the enzymes that makes LTB_4_, called 5-lipoxygenase (5-LO), could be detected on MVBs, and activated neutrophils released exosomes containing 5-LO from their trailing edges.

Exosomes purified from activated neutrophils contained high levels of 5-LO and other LTB_4_-synthesizing enzymes, and activation of resting neutrophils with fMLP led to an increase in the LTB_4_ concentration in exosomes. The authors also showed that the purified exosomes could mobilize resting neutrophils, inducing their polarization and adhesion, two key properties of activated, chemotaxing cells. The neutrophils migrated toward the exosomes, an effect that could be reduced significantly by treating the neutrophils with an antagonist for the LTB_4_ receptor.

These results clearly showed that exposure to a primary chemoattractant induced release of exosomes that contained and released LTB_4_, and that release of LTB_4_ by one neutrophil could promote chemotaxis in another neutrophil, a “paracrine” effect. But the authors found that LTB_4_ also had an autocrine effect, inducing stronger chemotaxis in the releasing cell as well. Knocking down two proteins critical for exosome docking and secretion, thereby reducing the amount of LTB_4_ released, led to a reduction in the directional specificity of and distance covered by the neutrophils containing the knockdowns, without affecting their speed of movement.

More work remains to be done to understand how LTB_4_ is released from the exosomes, whether through diffusion or transport or vesicle lysis. But this study makes it clear that the rapid and dramatic swarming of neutrophils to the site of an injury is mediated by release of exosomes carrying both LTB_4_ and the enzymes needed to synthesize it. These findings are likely to be directly relevant to developing treatments for several chronic inflammatory diseases, including asthma, in which LTB_4_ signaling plays an important role. The authors also suggest that exosome-mediated signaling is likely to be at work in other cell types and other situations requiring extracellular gradients that might otherwise be difficult to establish or maintain.

## Abbreviations

fMLP: N-formylmethionyl-leucyl-phenylalanine
LTB_4_: leukotriene B_4_
MVB: multivesicular body
5-LO: 5-lipoxygenase
